# Mitigating Aflatoxin B_1_-Induced Growth Impairment and Hepatic Stress in Nile Tilapia (*Oreochromis niloticus*): Comparative Efficacy of *Saccharomyces cerevisiae* and Silicate-Based Detoxifiers

**DOI:** 10.1007/s12602-023-10210-2

**Published:** 2024-01-04

**Authors:** Amr I. Zaineldin, Ehab Elsebaey, Ola A. Habotta, Walied S. Abdo, Mohammed F. El Basuini, Mahmoud A. O. Dawood

**Affiliations:** 1 Agriculture Research Center, Animal Health Research Institute (AHRI-DOKI), Kafrelsheikh, Egypt; 2 Department of Forensic Medicine and Toxicology, Faculty of Veterinary Medicine, Mansoura University, Mansoura, Egypt; 3 Department of Pathology, Faculty of Veterinary Medicine, Kafrelsheikh University, Kafrelsheikh, 33516 Egypt; 4 Faculty of Agriculture, Tanta University, Tanta, 31527 Egypt; 5 King Salman International University, El Tor, South Sinai, Nuweiba, 46618 Egypt; 6 Department of Animal Production, Faculty of Agriculture, Kafrelsheikh University, Kafrelsheikh, 33516 Egypt; 7 The Centre for Applied Research On the Environment and Sustainability, The American University in Cairo, Cairo, Egypt

**Keywords:** Aflatoxin B_1_, *Saccharomyces cerevisiae*, Silicate, Growth, Health, Nile tilapia

## Abstract

The objective of this study was to detect the effects of acute aflatoxin B_1_ (AFB_1_) exposure in Nile tilapia (*Oreochromis niloticus*) and the effectiveness of *Saccharomyces cerevisiae* and silicate in reducing these effects. Two hundred and forty Nile tilapia fingerlings (16 ± 0.5 g) were randomly assigned to four experimental groups, each with 60 fish and three replicates. Control basal diet (Diet 1) and three test diets were formulated, where Diet 2 was supplemented with 200 ppb AFB_1_. Diets 3 and 4 were intoxicated with AFB_1_ (200 ppb) and supplemented with 0.5% *S. cerevisiae* or 0.5%, respectively. After 60 days, Diet 1 had considerably greater growth characteristics than the other groups (*p* < 0.05). Diet 2 revealed a reduced (*p* < 0.05) survival rate after 1 month of exposure. In addition, Diet 1 showed higher (*p* < 0.05) total protein and albumin levels than Diets 3 and 4. AFB_1_ residues were detected in the liver in fish-fed Diet 2, Diet 4, and Diet 3. Alanine aminotransferase, aspartate aminotransferase, creatinine, and urea levels increased (*p* < 0.05) in fish-fed Diet 2. The glutathione peroxidase, lysozyme, and catalase activity were decreased (*p* < 0.05) in the fish-fed Diet 2. The malondialdehyde level was significantly higher in fish given Diet 2 (*p* < 0.05) than in fish-fed Diets 3 and 4. Histopathological investigation of fish-fed Diet 2 revealed impaired liver and spleen; however, both treatments (Diets 3 and 4) successfully lowered inflammation and preserved liver and spleen integrities. In conclusion, AFB_1_ impaired growth performance and posed a severe health risk to Nile tilapia. Furthermore, *S. cerevisiae* alleviated the contamination of AFB_1_ effects more efficiently than silicate employed for toxin adsorption.

## Introduction

Aquaculture is being expanded globally to offer food and nutritional security for the world’s rising population [1]. The initial step towards long-term aquaculture sustainability is a nutritionally balanced feed that is carefully manufactured and safe for nourishing aquatic species [1, 2]. Moreover, including plant protein components may pose significant concerns due to the possibility of fungal growth [3]. When certain physical, chemical, and biological conditions are present in uncontrolled storage conditions, fungi produce mycotoxins and toxic metabolites that accelerate the degradation of feed ingredients and feed [4]. To date, more than 300 distinct mycotoxins have been separated due to sophisticated methodologies and a growing interest in this field of research [5]. Aflatoxins (AF) constitute a significant class of mycotoxins, with aflatoxin B_1_ (AFB_1_) being the most toxic and displaying the most significant health risk [6]. AFB_1_ exposure may adversely affect aquatic animals’ health and performance [7]. Damage to the gills and intestines, failure of hepatorenal function, cytotoxicity, oxidative stress, neurotoxicity, and immunosuppression are the most obvious signs in aquatic animals fed diets contaminated with AFB_1_ [8, 9]. Accordingly, contaminated diets with AFB_1_ are the main reason for growth retardation in aquatic animals in fish farming. Thus, they result in high mortalities, reducing fish production and causing economic losses [9]. Fish suffering from aflatoxicosis B_1_ have pale gills, poor blood coagulation, slow growth, immunosuppression, and increased mortality [10]. Liver tumors are brought on by prolonged feeding of low quantities of AFB_1_, and they manifest as light-yellow lesions that can migrate to the kidney [10, 11].

Feeding Nile tilapia with diets polluted with aflatoxin in deficient concentrations can cause histopathological damage and disturb their physiological balance [12]. Residual aflatoxin levels in the liver and carcass of fish exposed to concentrations of aflatoxin higher than 350 µg kg^−1^ in the feed were previously detected, as well as exposure to 50 µg AFB_1_ kg^−1^ for 120 days in lambari (*Astyanax altiparanae*) fish which resulted in residual levels of AFB_1_ in the muscle tissue [13, 14]. Practically, destroying the contaminated feed is impossible; therefore, to avoid the effect of this toxic substance, improvement in animal immunity must be made. Various physical, chemical, and biological techniques are used to detoxify and decontaminate mycotoxin-containing animal diets. These techniques involve eliminating mycotoxins from contaminated feed ingredients, reducing the bioavailability of such mycotoxins in animal intestinal pathways, and directly destroying mycotoxins in the feed. Adsorbent-mediated AF degradation is a well-known and widely utilized approach for reducing the danger of mycotoxicoses in animals by integrating them into contaminated feed, thereby preventing and decreasing mycotoxicosis and transportation of mycotoxins into animal products [15–18]. Silica, or silicon dioxide (SiO_2_), is a primary binding agent in the food industry. It is also employed in the chemical sector as an adsorbent or glidant to allow powder to flow easily when producing tablets [19, 20]. Silica is not digested or absorbed in the intestinal tract, posing no toxicity risk. Moreover, silica is generally recognized as safe by the FDA [20].

Over the past 10 years, interest in the biological detoxification of AFB_1_ has grown significantly. According to several studies, lactic acid bacteria, bifidobacteria, and yeast can bind mycotoxins and minimize their toxicity [21–24]. *Saccharomyces cerevisiae* is the most effective probiotic for binding AFB_1_ in food and feed [25, 26]. Moreover, it is one of the biological biodegrading agents that can promote growth performance, immune activity, and carcass characteristics. The efficacy of *S. cerevisiae* cells, both intact and heat-inactivated, has been demonstrated as a toxin adsorbent [25, 27].

The present study aimed to study the effects of acute dietary AFB_1_ toxicity in Nile tilapia (*Oreochromis niloticus*) and to investigate the potential role of the probiotic yeast and silicate to ameliorate the health hazard effect of AFB_1_ in the diet of Nile tilapia. The growth performance, blood biochemistry, mortality rate, and bioaccumulation of aflatoxin residues were also highlighted.

## Materials and Methods

### Ethical Statement

The study protocol was approved by The Institutional Animal Care and Use Committee of Kafrelsheikh University’s ethical review board, Faculty of Agriculture, Kafrelsheikh University, Egypt.

### Fish and Experimental Facilities

A private farm in Kafr El Sheikh, Egypt, provided 240 mono-sex Nile tilapia (*O. niloticus*) fingerlings with a total initial body weight of 16 ± 0.5 g/fish. The fish were exposed to experimental conditions for 15 days in three indoor circular fiberglass tanks prior to the trial (1 m^3^). Fish were fed a control diet (30% crude protein and 6.5% crude fat) throughout this time. The fish were acclimated before being randomly divided into 12 glass aquariums, each measuring 30 × 40 × 60 cm. These aquariums represented four different types of experiments (in triplicate). There were 20 fish stocked in each tank. Throughout the experiment, feces and uneaten feed were removed from the aquarium by siphoning two-thirds of the water and replacing it with clean, well-aerated water from a storage tank. This allowed for the maintenance of clear, healthy water throughout the trial. During the 60-day feeding trial, fish were fed the test diets up to satiation level. Water quality measures were recorded throughout the experiment for temperature, pH, oxygen, salinity, and total ammonia nitrogen as follows: 27 ± 0.2 °C, 7.1 ± 0.1, 5.8 ± 0.1 mg/L, 11.5 ppt, and 0.1 ± 0.02 mg/L, respectively.

### Aflatoxin B_1_, *Saccharomyces cerevisiae*, and Silicate Sources

Aflatoxin was produced by growing *Aspergillus parasiticus* (standard toxigenic strain, NRRL 2999 culture, lyophilized strain, was kindly obtained from Vet. Med. Microbiology Dept., Iowa State University, USA) on rice fermentation. The moldy rice was steamed to kill the fungus, dried, milled, and analyzed for aflatoxin determination [28]. A *Saccharomyces cerevisiae*–based product (MiaMyco-Fit^®^: A product of MIAVIT GmbH Company, Germany) used in the study was added to the diet in a dose of 0.5 g/kg diet. Modified silicon dioxide (silicate) (Fylax^®^: A product of Selko Feed Additive) used in the study was added to the diet in a 0.5 g/kg diet.

### Test Diet Preparation

The basal diet was formulated using the ingredients in Table 1 with 30.1% crude protein and 17.9 kJ/kg gross energy. The first diet was assigned as control without any additives. The second, third, and fourth basal diets were supplemented with 200 ppb standard toxigenic strain of aflatoxin B_1_ (AFB_1_) as sublethal doses. The third diet was supplemented with 0.5 g MiaMyco-Fit^®^/kg as *S. cerevisiae*–based biological detoxifier. The fourth diet was supplemented with 0.5 g Fylax^®^/kg as a silicate-based detoxifier.

**Table 1:** Experimental diet formulation and proximate composition (% dry matter)

**Physical composition**	**Ingredient g/kg**			
	**Diet 1**	**Diet 2**	**Diet 3**	**Diet 4**
Fishmeal (67%)	60	60	60	60
Yellow corn (8%)	364	364	364	364
Dehulled-SBM (46%)^a^	440	440	440	440
Gluten (62%)	50	50	50	50
Wheat bran (12%)	50	50	50	50
Fish oil	10	10	10	10
Stay-C^7^	0.8	0.8	0.8	0.8
Vitamins mixture^b^	1	1	1	1
Minerals mixture^c^	1	1	1	1
NaCl	5	5	5	5
Lime	7	7	7	7
Mono-ca-phosphate	8	8	8	8
Sodium bicarbonate	1	1	1	1
Carboxymethyl cellulose	2	2	1.5	1.5
AFB_1_ (0.2 mg/kg)	0	200 (ppb)	200 (ppb)	200 (ppb)
MiaMyco-Fit/MiaBond^®^	0	0	0.5	0
Fylax-PLUS^®^	0	0	0	0.5
**Chemical composition**				
Dry matter	90.5	90.5	90.5	90.5
Crude protein	30.1	30.1	30.1	30.1
Ether extract	7	7	7	7
Crude fiber	8.35	8.35	8.35	8.35
Ash	7.5	7.5	7.5	7.5
Energy (KJg^−1^)*	17.9	17.9	17.9	17.9
Aflatoxins levels(µg kg^−1^)**	ND	215.4 ± 7	218.4 ± 7	211.8 ± 7

^*^Calculated using combustion values for protein, lipid, and carbohydrate of 236, 395, and 172 kJ g^−1^, respectively, and carbohydrate was calculated by the difference: 100 − (protein + lipid + ash)

^**^ND not detected

^a^Dehulled-SBM (46%): Dehulled soya bean meal, 46% crude protein

^b^Vitamin mixture (g/kg diet): β-carotene, 0.10; vitamin D3, 0.01; menadione NaHSO3∙3H2O (K3), 0.05; DL-α-tochopherol acetate (E), 0.38; thiamine-nitrate (B1), 0.06; riboflavin (B2), 0.19; pyridoxine–HCl (B6), 0.05; cyanocobalamin (B12), 0.0001; biotin, 0.01; inositol, 3.85; niacin (nicotinic acid), 0.77; Ca pantothenate, 0.27; folic acid, 0.01; choline chloride, 7.87; ρ-aminobenzoic acid, 0.38; cellulose, 1.92

^c^Mineral mixture (g/kg diet): MgSO4, 5.07; Na2HPO4, 3.23; K2HPO4, 8.87; Fe citrate, 1.10; Ca lactate, 12.09; Al (OH)3, 0.01; ZnSO4, 0.13; CuSO4, 0.004; MnSO4, 0.03; Ca (IO3)2, 0.01; CoSO4, 0.04

Ingredients and proximate compositions of the experimental diets are presented in Table 1. All ingredients were finely ground, weighed, mixed manually for 5 min, and then transferred to a mixer for another 15 min. After mixing the dietary ingredients in a food mixer, they were pelleted via a 1.6–2.1 mm diameter die in a laboratory storage container. The four formulated diets were prepared biweekly to ensure the intended AFB_1_ level. The pellets were air-dried at room temperature before being stored in a freezer until needed.

### Sample Collection

After 60 days, the fish was fasted for 24 h, and the following formulas were used to compute the growth parameters based on the body weight and length of each fish in each tank according to Bulut et al. [29]:

Weight gain (%) = (final weight-initial weight) × 100/initial weight.

Specific growth rate (SGR, %day^−1^) = [Ln (final weight) − Ln (initial weight)/duration] × 100.

Feed conversion efficiency (FCE) = live weight gain (g)/dry feed intake (g).

Protein efficiency ratio (PER) = live weight gain (g)/dry protein intake (g). Survival (%) = 100 × (final no. of fish/initial no. of fish).

### Whole-Body Proximate Analysis

Three individual fish were collected randomly from each replicate and kept at − 20 °C for later examination. All diets and whole-body fish composition, including dry matter, crude protein, crude fat, and ash content, were determined using AOAC [30] methodology. Viscera and liver of Nile tilapia were dissected from the fish above, weighted individually to calculate the viscerosomatic index (VSI) and hepatosomatic index (HSI) using the following formulae:

VSI (%) = weight of viscera/weight of fish × 100.

HSI (%) = weight of liver/weight of fish × 100.

### Biochemical Analysis

Blood samples were collected at the end of the experiment, and the serum was separated by centrifugation at 3000 rpm for 15 min at 4 °C. Serum samples were examined for the concentrations of total proteins and the major protein fractions in order to assess changes in the protein profile in Nile tilapia. To separate serum protein fractions, serum protein electrophoresis was carried out on agarose gel following the manufacturer’s application instructions using an automated electrophoresis machine and commercial diagnostic kits [28]. Zone electrophoresis was used on a buffered agarose gel with a pH of 8.7 to separate the various serum protein fractions.

The labeled sample wells on the agarose gel were filled with 10 µL of each serum sample. Each batch of samples contained control serum (Control Serum Human Normal, Sebia Corporate, France). The electrophoretic migration was carried out continuously for 15 min at 20 °C using 10 W, 40 mA, and 240 V. After migration, the gels were dyed in an amido black staining solution, then de-stained using acidic solutions and thoroughly dried. After the gels had been separated and stained, the staining intensity of each protein band was measured using a densitometer, the Epson Perfection V700 (Epson America Inc., CA, USA), together with the image analysis program Phoresis version 5.50 (Sebia Corporate, France).

Serum aspartate aminotransferase (AST), alanine aminotransferase (ALT), creatinine, and urea were detected using ready-made kits, following the manufacturer’s instructions [31].

### Aflatoxin Residue Analysis

Liver and muscle were collected from three fish per replicate, combined in triplicate, yielding three analytical samples per group, which were then transferred into individual microtubes for AFB_1_ identification and measurement. The tissues were ground and stored at − 20 °C until analysis. Michelin et al. [14] mentioned that aflatoxins were identified in liver and muscle samples using HPLC and immunoaffinity column cleanup.

### Histopathological Analysis

Three fish from each replicate tank were used for the liver and spleen morphology study. The entire spleen and liver were removed and collected to prepare the histological sample. All tissues were thoroughly washed with PBS (pH = 7.4) and immediately fixed in Davidson’s solution (agitated for 5 min) for 8 h to remove intestinal content. After that, the fixed tissues were gradually dehydrated in ethanol (70 to 100%), cleaned twice with xylene (1 and 2 h), and embedded in paraffin. Sections of 5-µm thickness were collected and stained with hematoxylin and eosin. Two cross-sectional slices were prepared from each tissue. The tissue slices were stained with hematoxylin and eosin before being examined using a light microscope (Eclipse 50i; Nikon, Tokyo, Japan) and camera (Digital Sight DS2MV with a DS-L2 control unit, Nikon) by using SigmaScan Pro 5 software. For each tissue, ten measurements were obtained according to de los Santos et al. [32].

### Measurement of Antioxidant Enzyme Activities

Using the thiobarbituric acid technique, malondialdehyde (MDA) levels were assessed [33]. The levels of GPx and catalase (CAT) in serum were assessed spectrophotometrically following the techniques used in other investigations [34] and [35], respectively. Lysozyme activity in serum was measured using turbidimetric assays [28]. The quantity of enzyme that resulted in a 0.001/min drop in absorbance was referred to as an enzyme activity unit.

### Statistical Analysis

Using SPSS version 22 (SPSS Inc., IL, USA), one-way analysis of variance was performed on the collected data. The homogeneity and normality of the variance were evaluated using the Shapiro–Wilk and Levene tests. Duncan’s test was used as a post hoc test, and differences between the means were examined at the 5% probability level.

## Results

### Growth Performance

The results of the growth and survival rate of *O. niloticus* exposed to either 200 ppb AFB_1_ with or without anti-mycotoxin (biological or chemical) treatments for 30 and 60 days are presented in Table 2. In comparison to the control group, all growth performances as indicated by the final weight (FW), weight gain (WG), specific growth rate (SGR), feed conversion efficiency (FCE), protein efficiency ratio (PER), and survivability were deteriorated significantly after 1 month feeding on 200 ppb aflatoxin B1, followed by chemical (silicate-based product) treated group (Diet 4) than biological (*S. cerevisiae*–based product) treated one (Diet 3) (*p* < 0.05), respectively.

**Table 2:** Growth performance and feed utilization parameters of Nile tilapia fed test diets for 60 days

**Duration**	**Parameters**	**Test diets**			
		**Diet 1**	**Diet 2**	**Diet 3**	**Diet 4**
Day 30	FBW (g)^a^	29.7 ± 0.6^c^	26.8 ± 0.7^b^	26.5 ± 0.5^ab^	24.9 ± 0.8^a^
	WG (%)^b^	84.3 ± 3.2^b^	66.3 ± 2.4^a^	68.7 ± 3.2^a^	56.8 ± 4^a^
	SGR^c^	0.9 ± 0.03^b^	0.78 ± 0.02^a^	0.8 ± 0.03^a^	0.69 ± 0.04^a^
	FCE^d^	1.09 ± 0.02	0.9 ± 0.03	1.03 ± 0.05	0.86 ± 0.08
	PER^e^	3.36 ± 0.07	2.8 ± 0.1	3.2 ± 0.15	2.66 ± 0.3
	Survival rate (%)	96.67 ± 3.33^b^	73.33 ± 3.33^a^	83.33 ± 7.3^ab^	86.67 ± 6.67^ab^
Day 60	FBW (g)	39.5 ± 0.5^c^	28 ± 0.6^a^	33.3 ± 1.5^b^	27 ± 0.6^a^
	WG (%)	145.4 ± 2.4^c^	73.9 ± 3.8^a^	111.9 ± 9.7^b^	70.1 ± 2.5^a^
	SGR	1.38 ± 0.02^c^	0.85 ± 0.03^a^	1.15 ± 0.07^b^	0.8 ± 0.02^a^
	FCE	0.83 ± 0.01^c^	0.42 ± 0.03^a^	0.7 ± 0.5^b^	0.46 ± 0.01^a^
	PER	2.56 ± 0.03^c^	1.3 ± 0.08^a^	2.14 ± 0.1^b^	1.4 ± 0.04^a^
	Survival rate (%)	91.67 ± 3.3^c^	61.67 ± 3.3^a^	78.33 ± 7.3^bc^	66.67 ± 1.67^ab^

Values are means of triplicate groups’ ± SEM. Within a row, means with the same superscript letters are not significantly different (*p* > 0.05)

^a^FBW: final body weight (g)

^b^WG: percent weight gain (%)

^c^SGR: specific growth rate (% day^−1^)

^d^FCE: feed conversion efficiency

^e^PER: protein efficiency ratio

### Whole-Body Proximate Analysis

The body composition and somatic indicators of Nile tilapia are shown in Table 3. Except for whole-body protein and hepatosomatic index (*p* > 0.05), investigated diets demonstrated non-significant (*p* > 0.05) effects on whole-body proximate analysis and somatic indices when compared to the control group. Diet 2-fed fish had the lowest whole-body crude protein content and HSI (*p* > 0.05).

**Table 3:** Whole-body proximate analysis and somatic parameters in Nile tilapia fed tested diets for 60 days

**Items**	**Test diets**			
	**Diet 1**	**Diet 2**	**Diet 3**	**Diet 4**
Dry matter	30.5 ± 0.2	29.5 ± 0.2	31.4 ± 0.2	30.1 ± 0.2
Moisture	69.5 ± 0.5	70.5 ± 0.2	68.6 ± 0.5	69.9 ± 0.3
Crude protein	16.2 ± 0.3^b^	14.5 ± 0.3^a^	15.6 ± 0.3^ab^	15.25 ± 0.3^ab^
Crude lipid	7.1 ± 0.2	5.8 ± 0.2	6.8 ± 0.2	6.5 ± 0.2
Crude ash	5.5 ± 0.4	6.3 ± 0.4	5.6 ± 0.4	5.5 ± 0.4
VSI (%)	10.4 ± 0.6	10.1 ± 0.6	10.1 ± 0.6	10.3 ± 0.6
HSI (%)	3.4 ± 0.1^b^	2.4 ± 0.18^a^	3.1 ± 0.15^b^	2.7 ± 0.12^a^

Values are means of triplicate groups ± SEM. Within a row, means with the same letters are not significantly different (*p* < 0.05)

^a^VSI: viscerosomatic index = 100 × viscera weight/fish weight

^b^HSI: hepatosomatic index = 100 × liver weight/fish weight

### Aflatoxin B_1_ Residues

Only the liver of Nile tilapia fish was substantially (*p* < 0.05) affected by the experimental treatments; the muscular tissues remained unaffected (Table 4). Aflatoxin accumulation in the liver of fish given 200 ppb aflatoxin B1–contaminated diet (AFB_1_) (Diet 2) rose considerably (*p* < 0.05). However, after supplementation with chemical (silicate-based product) or biological (*S. cerevisiae*–based product) treatments considerably (*p* < 0.05) lowered the concentration of aflatoxin residues, with an advantage towards the biological treated group (*S. cerevisiae*–based product) (Diet 3). Furthermore, no residues were discovered in fish muscle in all fish groups fed on the experimental diets for 60 days.

**Table 4:** AFB_1_ accumulation (µg kg^−1^) in liver and muscle tissue of Nile tilapia fed test diets for 60 days

	**Tissue**	Diet 1	Diet 2	Diet 3	Diet 4
	**Liver**				
Day 30		ND	2.13 ± 0.04^b^	1.45 ± 0.03^a^	1.71 ± 0.08^ab^
Day 60		ND	3.27 ± 0.03^b^	1.57 ± 0.03^a^	2.5 ± 0.03^ab^
	**Muscle**				
Day 30		ND	ND	ND	ND
Day 60		ND	ND	ND	ND

Data represent means ± pooled SEM. Values with different letters are significantly different (*p* < 0.05). Values with the same letter are not significantly different (*p* > 0.05)

*ND* not detected

### Liver and Spleen Histopathological Analysis

**Fig. 1:**
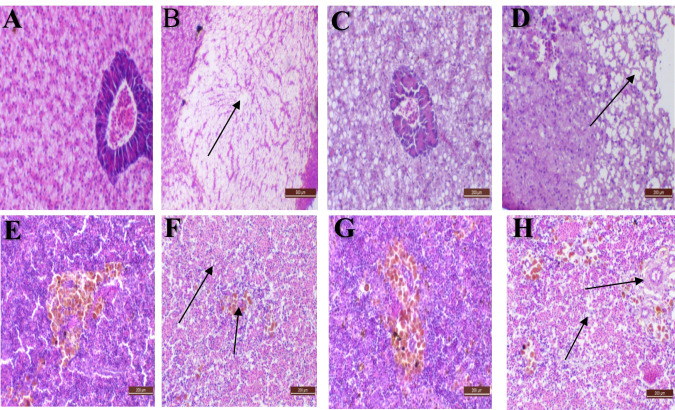
**A** The liver of control Nile tilapia (*Oreochromis niloticus*) showed both normal hepatocytes and hepatopancreas (H&E, X 200). **B** The liver of fish supplied with AFB_1_ alone showed clear cell type preneoplastic foci, loss of regular structure. **C** The liver of fish supplied with AFB_1_ and treated with (MiaMyco-Fit^®^) was within the normal limits (H&E, bar = 200 µm). **D** The liver of supplied with AFB_1_ and treated with (Fylax^®^) showed liver degeneration signs and loss of hepatocytes regular structure. **E** The spleen in the control group demonstrates a normal structure. **F** Fish administered with AFB_1_ alone had depleted lymphocytes and lacked a melano-macrophage core in their spleen. **G** Spleen of biologically treated fish was within the normal structure limits. **H** Spleen of chemically treated fish showed lymphoid depletion similar to aflatoxicated group

The most visible pathologic lesions were found in fish livers given a 200 ppb AFB_1_-contaminated diet (Diet 2). The livers had significant vacuolation, hepatocyte degradation, and necrotic alterations in the hepatopancreas. There were several preneoplastic foci as eosinophilic and clear foci types and significant nodular pancreatic proliferation (Fig. 1B). The liver of the control fish showed both normal hepatocytes and hepatopancreas. The liver of fish supplied with AFB_1_ and treated with *S. cerevisiae*–based product was within the normal limits (Fig. 1C): The liver supplied with AFB_1_ and treated with silicate showed liver degeneration signs and loss of hepatocytes regular structure (Fig. 1D). The spleen of fish given an AFB_1_-contaminated diet (200 ppb) without any treatment showed considerable lymphoid hypoplasia, severe atrophy of the melano-macrophage centers, and red pulp congestion (Fig. 1F). Spleens in the group given AFB_1_-contaminated diet but treated with silicate feed additive showed lymphoid hypoplasia with somewhat less exaggeration where lymphocytes occurred in lymphoid follicles; however, spleen of fishes fed on AFB_1_-contaminated diet and treated with *S. cerevisiae*–based product showed marked lymphocytes within the white pulp and normally scattered melano-macrophages centers (Fig. 1G, H).

### Blood Serum Protein

**Fig. 2:**
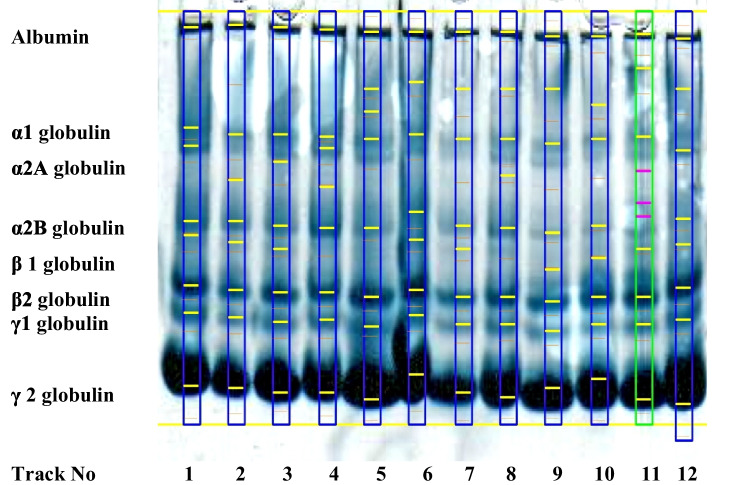
Photograph of SDS-PAGE of the protein fractions of Nile tilapia (*Oreochromis niloticus*) in control group (Track No; 7, 8 and 10) and the experimental groups; Aflatoxicated fish (Track No; 1, 3, and 9), Biologically treated fish (Track No; 4, 5 and 12), and Chemically treated fish (Track No; 2, 6, and 11). From left to right (1–8), the sections comprise albumin, alpha-1, alpha-2A, alpha-2B, beta-1, beta-2, and gamma-1 and gamma-2 globulins

**Fig. 3:**
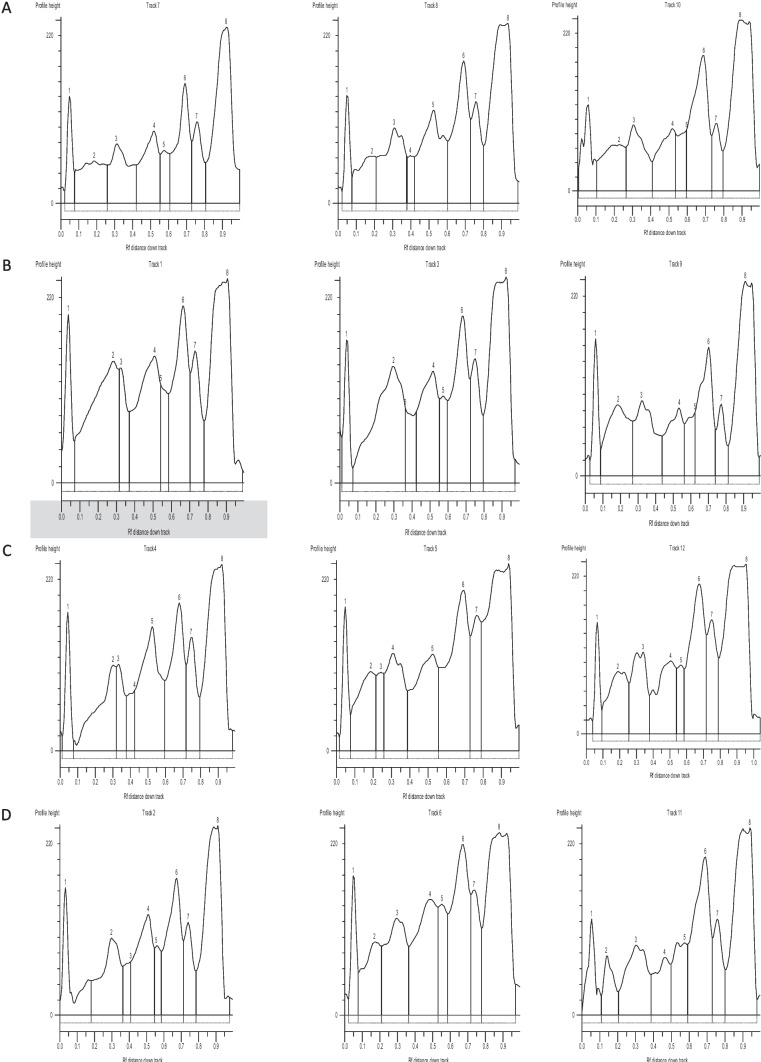
Electrophoretogram of serum protein of Nile tilapia (*Oreochromis niloticus*) in control group (**A**) and the experimental groups (**B** aflatoxicated fish, **C** biologically, and **D** chemically treated). From left to right (1–8), the sections comprise albumin, alpha-1, alpha-2A, alpha-2B, beta-1, beta-2, and gamma-1 and gamma-2 globulins

Figure 2 reveals a photograph of SDS-PAGE of the protein fractions in the serum of Nile tilapia. Figure 3 demonstrates the electrophoretograms of the protein fractions and changes in their proportion in all tested groups. Following the addition of 200 ppb AFB_1_ to the Nile tilapia diet (Diet 2), total protein and albumin were considerably (*p* < 0.05) decreased, while gamma one globulin levels were markedly increased (Table 5 and Figs. 2 and 3). Furthermore, when compared to the (Diet 2) group, the chemical and biological treatments significantly (*p* < 0.05) increased the levels of total protein and albumin and reduced gamma one globulin level to mimic the values in the control.

**Table 5:** Blood total protein, albumin, and globulin in Nile tilapia fed test diets for 60 days

**Parameters**	**Diet 1**	**Diet 2**	**Diet 3**	**Diet 4**
Total protein	2.7 ± 0.04^b^	2.57 ± 0.03^a^	2.67 ± 0.01^ab^	2.68 ± 0.04^ab^
Albumin	0.89 ± 0.01^b^	0.72 ± 0.02^a^	0.85 ± 0.02^b^	0.83 ± 0.02^b^
α1 globulin	0.2 ± 0.03	0.2 ± 0.02	0.24 ± 0.007	0.21 ± 0.003
α2A globulin	0.41 ± 0.006	0.37 ± 0.01	0.43 ± 0.03	0.45 ± 0.03
α2B globulin	0.26 ± 0.12	0.12 ± 0.01	0.35 ± 0.13	0.17 ± 0.04
β1 globulin	0.22 ± 0.08	0.36 ± 0.06	0.24 ± 0.07	0.35 ± 0.08
β2 globulin	0.39 ± 0.02	0.25 ± 0.09	0.21 ± 0.07	0.32 ± 0.1
γ1 globulin	0.21 ± 0.03^a^	0.43 ± 0.05^b^	0.24 ± 0.02^ab^	0.2 ± 0.06^a^
γ 2 globulin	0.12 ± 0.01	0.13 ± 0.01	0.1 ± 0.01	0.15 ± 0.03

Data represent means ± pooled SEM. Values with different letters are significantly different (*p* < 0.05). Values with the same letter are not significantly different (*p* > 0.05)

### Serum Biochemical Evaluations

Compared to the control group (group 1), Nile tilapia exposed to 200 ppb AFB_1_ (Diet 2) had substantially higher serum AST and ALT hepatic enzyme activity, as shown in Table 6. These levels were also markedly reduced within the chemical or biological treated groups (Diet 3 and Diet 4). With increasing exposure period, from 30 to 60 days, these values within each group also considerably rose.

**Table 6:** Liver enzyme activity of Nile tilapia fed test diets for 60 days

**Parameters**	**Diet 1**	**Diet 2**	**Diet 3**	**Diet 4**
ALT^a^	2.21 ± 0.08^a^	4.34 ± 0.11^c^	2.69 ± 0.1^a^	3.45 ± 0.12^b^
AST^b^	61.67 ± 0.96^a^	97.73 ± 4.2^b^	67.37 ± 1.4^a^	70.73 ± 2.8^a^

Data represent means ± pooled SEM. Values with different letters are significantly different (*p* < 0.05). Values with the same letter are not significantly different (*p* > 0.05)

^a^ALT: alanine amino transferase

^b^AST: aspartate aminotransferase

After 30 days of the trial, exposure to AFB_1_ (Diet 2) significantly increased serum creatinine and urea levels (Table 7). The effect of 200 ppb AFB_1_ (Diet 2) on Nile tilapia’s serum creatinine and urea levels was interestingly and significantly reduced by biological and chemical treatments (Diets 3 and 4, respectively). Furthermore, the biological treatment (Diet 3) revealed higher efficacy in decreasing the creatinine and urea levels, similar to the control group after 60 days of the trial.

**Table 7:** Kidney function indicators in Nile tilapia fed test diets for 60 days

**Parameters**	**Diet 1**	**Diet 2**	**Diet 3**	**Diet 4**
Creatinine (mg/dl)	0.28 ± 0.01^a^	0.46 ± 0.01^b^	0.30 ± 0.01^ab^	0.32 ± 0.01^ab^
Urea (mg/dl)	3.21 ± 0.02^a^	4.25 ± 0.05^b^	3.34 ± 0.07^ab^	3.54 ± 0.03^ab^

Data represent means ± pooled SEM. Values with different letters are significantly different (*p* < 0.05). Values with the same letter are not significantly different (*p* > 0.05)

### Antioxidative‐Related Biomarkers

**Fig. 4:**
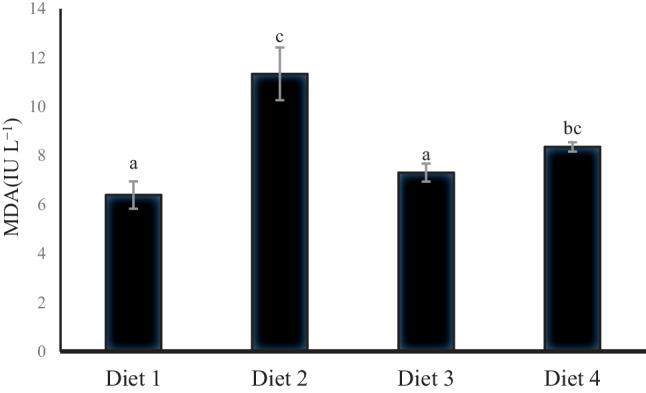
Malonaldehyde activity (IU L.^−1^) of Nile tilapia fed test diets. Values are expressed as mean ± SE from triplicate groups. Values with different letters are significantly different (*p* < 0.05). Values with the same letter are not significantly different (*p* > 0.05)

**Fig. 5:**
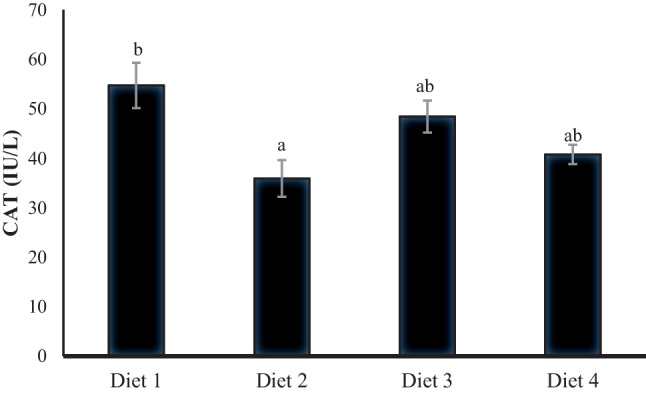
Catalase activity (IU/L) of serum in Nile tilapia fed test diets. Data represent means ± pooled SEM. Values with different letters are significantly different (*p* < 0.05). Values with the same letter are not significantly different (*p* > 0.05)

**Fig. 6:**
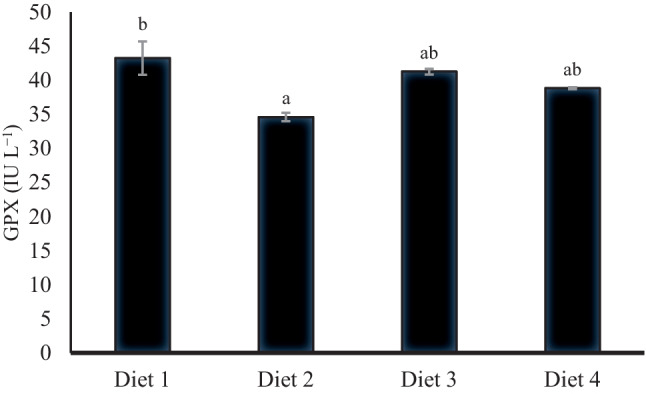
Glutathione peroxidase (IU L.^−1^) of Nile tilapia fed test diets. Values are expressed as mean ± SE from triplicate groups. Values with different letters are significantly different (*p* < 0.05). Values with the same letter are not significantly different (*p* > 0.05)

**Fig. 7:**
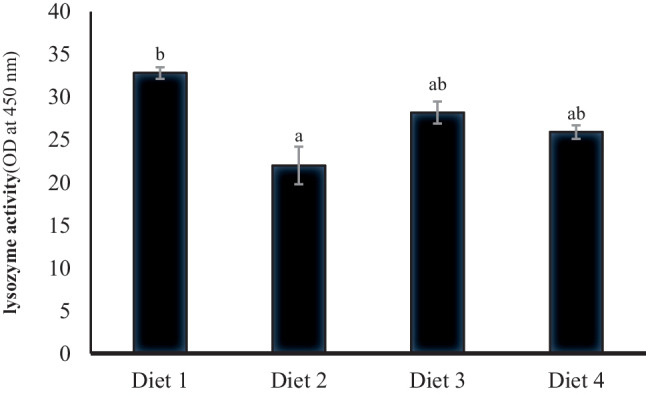
Lysozyme activity (OD at 450 nm) of serum in Nile tilapia fed test diets. Data represent means ± pooled SEM. Values with different letters are significantly different (*p* < 0.05). Values with the same letter are not significantly different (*p* > 0.05)

Figures 4, 5, 6, and 7 demonstrate the antioxidative and immune parameters in tested fish. The serum MDA activity increased significantly (*p* < 0.05) in fish fed 200 ppb AFB_1_ (Diet 2) compared to other tested groups. Moreover, it showed significantly declined serum catalase, glutathione peroxidase, and lysozyme activities compared to the control group (*p* < 0.05; Figs. 5, 6, and 7). *S. cerevisiae*–based product (MiaMyco-Fit) enhanced all antioxidant and immune parameters more efficiently than the silicate-fed group.

## Discussion

As a result of the growing usage of plant-based additives in aquaculture feeds, aquatic animals became more sensitive to the effects of mycotoxins [28, 36]. Aflatoxin is a prevalent mycotoxin that has a deleterious impact on the health and development of aquatic animals. Aflatoxin B_1_ (AFB_1_) is a biological toxin generated by *Aspergillus flavus* and *Aspergillus parasiticus* when environmental conditions are appropriate [37]. These toxic substances can impact animal health and production, as well as cause harm to human beings if they are ingested [28, 36, 37]. The present study employed acute AFB_1_ toxication (200 ppb) for Nile tilapia with or without medications. Exposure of Nile tilapia to AFB_1_ at 200 ppb for 8 weeks has significantly reduced growth performance, including total weight gain, specific growth rate, protein efficiency ratio, and survivability, compared to control. These results were consistent with previous findings revealing similar growth performance retarding effects of aflatoxin in Nile tilapia, thinlip grey mullet (*Liza ramada*), and rainbow trout (*Oncorhynchus mykiss*) [9, 38–41]. The reduction of survivability of tilapia by AFB_1_ that has been reported is similar to what previously demonstrated in a 200 ppb/kg AFB_1_ in diet, fed for 10 weeks or 16 weeks, showed 34.34% and 30% mortality rate in Nile tilapia, respectively [28, 42]. The observed decrease in growth performance and fish survival might be related to protein synthesis impairment, poor hepatic metabolism, suppressed appetite, and nutritional malabsorption [38, 43].

Administration of yeast and silicate attenuated the detrimental effects of mycotoxins on weight gain and FCR of fish as suggested by the previous studies [42, 44–46]. In this study, the addition of *Saccharomyces cerevisiae*–based diet significantly counteracted the growth-suppressing effects of AFB_1_. At the same time, supplementation of Nile tilapia with silicate did not succeed in similar growth improvement. The success of yeast in boosting the growth and survivability of Nile tilapia may be related to its supportive effects on internal organs, particularly liver integrity, which is the first organ to be harmed by AFB_1_ [44, 47]. Similarly, Rahman et al. [48] reported that *S. cerevisiae* relieved the negative impacts of AFB_1_ exposure and enhanced the growth performance of Nile tilapia. In addition, Yang et al. [49] reported that turbot (*Scophthalmus maximus*) intoxicated with AFB_1_ and treated with yeast cell wall extract showed enhanced growth performance and feed efficiency. Yang et al. [49] related the mitigation role of yeast cell wall extract on AFB_1_ toxicity with the promoted intestinal microbiota diversity and reduced abundance of harmful pathogens. The liver is the main organ for silicate metabolism [50]; therefore, the lower growth value of the silicate-based product in this trial may be due to the aflatoxin-degenerative effect on the fish liver. HSI fish morphological indicator provided information on the physiological state and nutritional health of the fish. The loss of vacuolation and fat in the liver probably caused the significant decline in HSI in fish-fed Diet 2.

Mycotoxins present in fish directly endanger human health, primarily because of their toxic effects. Since there is currently no safe level for mycotoxin residues in fish, detecting mycotoxins in fish organs and tissues is crucial for determining the danger to public health and the amounts of mycotoxins in various fish tissues. AFB_1_ accumulation in the hepatic and muscular tissue was demonstrated in several studies [51–55]. In most of these trials, the residual AFB_1_ level in the liver is more significant than in the muscle. In this investigation, the AFB_1_ residue was discovered only in the liver of Nile tilapia fed on tested diets but not in the edible flesh. The most likely reason for that is the presence of a variety of P450 enzymes in the liver that play an essential role in the AFB_1_ metabolism and detoxification process, in which AFB_1_ is activated to the toxic form (AFBO) or converted to be less toxic as AFM_1_, AFP_1_, and AFQ_1_ [56]. This finding lines up with previous tilapia research, indicating that it is probably safe to ingest just the edible flesh of tilapia even when fed diets contaminated with a somewhat high AFB_1_ level [57, 58]. Supplementation with a *S. cerevisiae*–based product significantly reduced AFB_1_ accumulation in the liver, while silicate did not as compared to the AFB_1_-intoxicated group. This might be related to yeast’s involvement in improving liver function and maintaining hepatic cell integrity, which enhances AFB_1_ metabolism in liver tissue [47]. For the protection of human beings, detailed knowledge of the bioaccumulation of aflatoxins and their metabolites in aquatic species is required. Consequently, there is a need for scientific studies linking the amounts of AFB_1_ in the diet to the quantities of fish tissue intended for human consumption.

Serum protein concentrations in fish vary based on the species, age, and health of the fish. Healthy fish have greater blood protein concentrations than unhealthy fish [59]. Stressful environmental factors, such as high temperatures or low oxygen levels, can also alter blood protein concentrations in fish [60]. The gamma area, mostly made up of antibodies of the IgG type, is the region that receives the most attention in the development of serum protein ionophoresis [61]. Many conditions, such as connective tissue disorders, liver cirrhosis, and chronic infections (granulomatous diseases), may cause an increase in the gamma-globulin zone [62]. In this study, AFB_1_ poisoning reduced total blood protein and albumin levels while increasing gamma globulin levels (Table 4, Figs. 2 and 3), indicating liver tissue injury [63]. Normal levels of total protein, albumin, and even globulin fractions were found in yeast and silicate-supplemented groups, indicating a positive impact on the liver tissue, health, and its function.

Liver enzyme activity (AST and ALT) was markedly increased in the case of the AFB_1_-supplemented group and returned to normal levels with yeast supplementation. Expectedly, previously mentioned proof of liver injury revealed after AFB_1_ exposure might contribute to releasing hepatic enzymes (AST, ALT, and ALP) in the blood circulation, raising their serum levels. Similarly, Nile tilapia [57], sea bass (*Dicentrarchus labrax*) [53], turbot [49], and gibel carp [54] intoxicated with AFB_1_ revealed deteriorated secretion of AST and ALT. Further, Eraslan et al. [64] showed considerable hepatocellular deterioration together with elevated hepatic enzyme activity, demonstrating a severe impairment of liver function following AFB_1_ intoxication in adult Wistar albino rats. In this study, the normal levels of liver enzyme activity demonstrated in the case of the yeast supplementation group may be due to the impact of yeast and its different components (mannan-oligosaccharide and β-glucan) on hepatocytes integrity and health. This supports earlier studies that found dietary *S. cerevisiae* dramatically improved liver health and function [65–67]. Dietary AFB_1_ was demonstrated to cause renal damage, which may entail inflammation, cell necrosis, and toxicosis based on the biochemical measures in this study that showed higher serum creatinine and urea concentrations.

Together with those mentioned above, the current findings highlighted the adverse effects of AFB_1_ on liver and spleen histomorphology of Nile tilapia, showing clear cell type preneoplastic foci, loss of regular structure of hepatic cells, and depleted lymphocytes and lacked a melano-macrophage core in their spleen [28]. After *S. cerevisiae* supplementation, these adverse effects were successfully alleviated, confirming the *S. cerevisiae’s* positive benefits on the health of the liver’s tissues, hepatocytes, and fish spleen. This histologically positive yeast effect on liver and spleen histomorphology is a marker of improved antioxidative status of Nile tilapia following supplementation with *S. cerevisiae*–based product (MiaMyco-Fit) as narrated by previous studies [28, 68–72]. In this regard, Pinheiro et al. [73] also reported that tambaqui (*Colossoma macropomum*) intoxicated with AFB_1_ exhibited deteriorated liver histological features; however, dietary *S. cerevisiae* relieved the negative impacts of AFB_1_ on the liver tissue. AFB_1_ is metabolized, detoxified, and/or conjugated with nucleic acids and proteins in the liver, which is its target organ [74]. The liver, muscle, and other edible animal tissues have a certain amount of aflatoxin that may accumulate over time without causing any alterations [75]. Numerous studies have shown that animals exposed to AFB_1_ have residues in their tissues that induce negative health consequences when consumed [28, 53, 76]. In the current study, there were no measurable residual amounts of AFB_1_ in the muscles of any group. It was shown that Nile tilapia fed diets containing AFB_1_ alone, without treatment, had significant residual quantities of the toxin in their livers. This finding is consistent with other studies on tilapia exposed to long-term dietary aflatoxin [57]. However, adding *S. cerevisiae* significantly reduced those levels, whereas silicate did not. This result is similar to previous research that gave broiler chicken liver diets containing AFB_1_ (100 g/kg) and the yeast *Pichia kudriavzevii* (0.1%); there was a drop in the residual levels of AFB_1_ [44].

Oxidative stress is a potential danger factor for livestock development because it can produce large amounts of reactive oxygen species (ROS), resulting in oxidant activity that surpasses antioxidant neutralization capacities and impaired antioxidant system effectiveness [77, 78]. Malondialdehyde (MDA), lysozyme, glutathione peroxidase (GPX), and catalase activity (CAT) in Nile tilapia serum were examined in the current study using ELISA. It was discovered that MDA was considerably higher and that lysozyme, GPX, and CAT were significantly reduced in the group that had received AFB_1_ without treatments. MDA is a byproduct of the peroxidation of polyunsaturated fatty acids. It has been employed as a biomarker to assess oxidative stress [79, 80]. The increased MDA activity indicates that Nile tilapia given AFB_1_-contaminated feed are under considerable oxidative stress [81]. Furthermore, given an aflatoxicated diet with yeast and silicate administration, Nile tilapia had lower MDA levels that were substantially identical to the control group. Many studies have demonstrated that *S. cerevisiae* is a practical, viable, and low-cost tool for minimizing oxidative stress, is one of the potential approaches to lowering the risk associated with the presence of mycotoxins in feedstuffs, and can be regarded as a biological detoxifier [22, 25, 82–84].

Catalase is an essential antioxidant enzyme that plays a vital role in the H_2_O_2_ scavenging process by converting H_2_O_2_ to H_2_O and O_2_ [85]. The reduced CAT level in the AFB_1_-fed group without treatments indicates the negative impact of AFB_1_ on the antioxidant defense system. Nile tilapia–fed AFB_1_ supplemented diet with *S. cerevisiae*–based product succeeded in relieving the reduced effect on CAT activity even more than the silicate group. In order to diminish the hydrogen peroxide’s adverse effects, the intracellular antioxidant enzyme glutathione peroxidase-1 (GPx-1) transforms superoxide anion radicals to hydrogen peroxide, protecting cells from oxidative damage [86]. Similarly, AFB_1_ resulted in a lower activity of GPX in Nile tilapia. In contrast, *S. cerevisiae* and silicate have improved GPX levels, indicating the positive effects of biological and chemical detoxifiers used in this study on the Nile tilapia antioxidant system. In line with the present study, AFB_1_ exposure induced oxidative damage in Nile tilapia [87], northern snakehead (*Channa argus*) [88], and thinlip grey mullet [38], while supplementary lipopolysaccharides and probiotics mitigated AFB_1_-negative impacts on the antioxidative capacity in Nile tilapia [87] and thinlip grey mullet [38].

Lysozyme is a vital enzyme of leucocytic origin that is a crucial component of the innate immune system’s defense against microbial invasion (bacteria, viruses, and fungi) [89]. Hereby, AFB_1_ demonstrated a reduction in the lysozyme secretion, which directly impacts Nile tilapia’s immune status. This negative impact was relieved significantly after supplementation with *S. cerevisiae* and silicate-based products. The improved antioxidative and immune status previously mentioned in the case of yeast administration might be related to the cell components of *S. cerevisiae* since the cell wall is mainly made up of an inner layer of β-glucans and chitin and an outer layer of highly glycosylated mannoproteins, which have a substantial influence on toxin adsorption efficacy in *S. cerevisiae* [82]. In addition, silicate has been recorded as a potent toxin adsorbent in several investigations, and the favorable impacts displayed on antioxidation, and immunological boosting mechanisms are because of the silicate capacity in the mycotoxin adsorption process [90–95].

## Conclusion

In the present study, the effect of aflatoxin on Nile tilapia was investigated. Further, the protective influence of the biological and chemical treatments against the toxic action of AFB_1_ was also estimated. The AFB_1_ drastic effects on growth, survivability, and immunity of Nile tilapia were demonstrated. The biological treatment (*S. cerevisiae*) has significantly decreased the adverse effects of AFB_1_ on Nile tilapia throughout, supporting the hepatocytes integrity more than the silicate-treated group.

## Data Availability

The authors confirm that the data supporting the findings of this work are available within the article. Raw data that supports the findings are available upon reasonable request.
